# Three Kinds of Nonconceptual Seeing-as

**DOI:** 10.1007/s13164-017-0339-2

**Published:** 2017-05-26

**Authors:** Christopher Gauker

**Affiliations:** Department of Philosophy, Faculty of Cultural and Social Sciences, University of Salzburg, Franziskanergasse 1, 5020 Salzburg, Austria

## Abstract

It is commonly supposed that perceptual representations in some way embed concepts and that this embedding accounts for the phenomenon of seeing-as. But there are good reasons, which will be reviewed here, to doubt that perceptions embed concepts. The alternative is to suppose that perceptions are *marks* in a perceptual similarity space that map into locations in an objective quality space. From this point of view, there are at least three sorts of seeing-as. First, in cases of ambiguity resolution (such as the duck-rabbit), the schematicity of the figure leaves us with a choice as to where in perceptual similarity space to place a mark (closer to the marks that represent rabbits or closer to the marks that represent ducks). Second, in cases where expertise affects perception (as when, for example, we learn to distinguish various kinds of tree leaves), the accumulation of perceptual landmarks permits a more precise placement of a mark in perceptual similarity space. Third, extensive experience with an object (e.g., the family dog) allows similarity to that object to serve as an acquired dimension in perceptual similarity space, which in turn affects the relative similarities of other objects.

## Introduction

What happens in your mind when you see the duck-rabbit as a duck?^1^ And what happens when you switch from seeing it as a duck to seeing it as a rabbit? It is tempting to answer in terms of concepts. When one sees the duck-rabbit as a duck, the concept *duck* is built into one’s very perception of the drawing. When one switches to seeing it as a rabbit, one’s perception sheds the concept *duck* and takes on the concept *rabbit*.

What happens when one acquires the ability to distinguish between various kinds of tree leaves just by looking at them? At first, one could not reliably distinguish between the leaves of a beech tree and the leaves of an elm tree, but then, later, one could. It is tempting to answer that one’s perceptions come to be informed by concepts of the various kinds of leaves. It comes to pass that the concept *beech leaf* is built into one’s very perception of the beech leaf.

Thus, these different phenomena of seeing-as (also called *aspect perception*) may be taken to demonstrate the presence of concepts in perceptual representations. I will argue that there is a better way to conceive of the various phenomena of seeing-as. Perceptions are best conceived as *marks* in a *perceptual similarity space* that correspond to *locations* in an *objective quality space*. The relative locations in perceptual similarity space of the marks representing three objects can be taken as a measure of the perceived similarity of these three objects relative to one another. On this account, the various phenomena of seeing-as are distinct, but each brings to light an aspect of the recording of marks in perceptual similarity space. I will countenance three such phenomena, which I will call *ambiguity resolution*, *heightened discrimination* and *dimension addition*. But first, I will explain what I mean by *concepts* and then I will explain why I deny that concepts are built into perceptions.

## What Concepts Are

The traditional conception of concepts is bound up with the idea that in speaking we express our thoughts. The process of communication supposedly begins when a speaker has a thought in mind that he or she aims to reveal to a hearer. The thought, together with a motive to express it, somehow issues in speech. The thought can be described has having a *content*, and communication is successful when the hearer, as a consequence of hearing the speaker’s speech, comes to grasp the content of the speaker’s thought. Grasping the content could take the form of believing what the speaker believes or simply recognizing that the speaker has a thought with that content. For instance, if the speaker utters the (German) words, “Es gibt Säugetiere, die Eier legen,” he or she may thereby express the thought with the content that *some mammals lay eggs*. The communication is successful if the hearer, on the basis of understanding the words spoken, learns that the speaker thinks that *some mammals lay eggs*.^2^

In light of this theory of linguistic communication, concepts can be characterized as follows:

- Concepts are the building blocks of the kinds of thoughts expressed in sentences, which stand to whole thoughts as words stand to whole sentences.

So if the thought expressed with the words, “Es gibt Säugetiere, die Eier legen” is the thought that *some mammals lay eggs*, then one of the concepts in that thought will be the concept *mammal*, which is expressed by the word “Säugetier”.

Having said that much, we can still conceive of concepts in two different, but entirely compatible ways. First, we can think of concepts as components of the concrete tokens of thoughts in the brain. The thought itself might have a physical structure corresponding more or less to the physical structure of the sentence used to express it, and concepts might be conceived as components, or aspects, of this structure. Secondly, we can think of concepts as components of the *content* of the thought expressed. According to the underlying theory of communication, speakers are able to succeed in communicating the contents of their thoughts to hearers, because the words they speak have a content too, which speaker and hearer both attach to the words spoken. So concepts in one sense are the components of shared content that correspond to individual words and phrases. (If we go along with the commonplace idea, espoused for instance in Kaplan 1989, that propositional contents are structures built up out of individuals and properties, then concepts in this sense might turn out to be individuals or properties, which, though odd terminologically, is a consequence we can get used to.) Although I think it is fair to explicate the concept *concept* in this way, this explication does not at all imply that only creatures with language have concepts. Even if a creature does not have language, it might possess the kinds of thoughts that could be expressed in speech if only it had a suitable language.

In what follows, when I speak of concepts, I will be treating them as components of content. What I want to argue is that concepts are not components of the representational content of perceptions. If I am right about that, then there will no longer be any reason to think of concepts as components or aspects of token perceptions in the mind/brain either, since the only reason to attribute conceptual structure to token perceptions would be to accommodate the idea that perceptions have concepts as contents.

## Which Concepts?

If perceptions have conceptual contents, then it should be possible sometimes to *say* what the conceptual content of any given perception is. I do not say that it should be possible to *figure out* the conceptual content of a perception is, because I do not want to imply that it should be so hard that one has to “figure it out”. I do not say that it should *always* be possible to say what the content is; the perception is quickly gone and one may have forgotten. However, there should be perceptions of which we can say with some certainty what their conceptual content is. A doubt that one can raise against the thesis that perceptions have conceptual content is whether it is *ever* possible to say what the conceptual content of a perception is. I cannot prove that it is never possible to do that. But I can sharpen the challenge by examining some options. The objective of this exercise is not refutation but just to raise enough doubt to clear a space for a very different conception of the manner in which perceptions represent, which I will then present in the next section.

Anyone who aims to identify the conceptual content of a given perception has to make a choice between two basic approaches:

- *The high road:* The concepts that compose the contents of perception include some *general* concepts.
- *The low road:* The concepts that compose the contents of perception are exclusively *maximally specific* concepts.

By a *maximally specific concept* I mean a concept that is not divisible into species. For example, the concept *rectangular* is not maximally specific because there are various specific rectangular shapes, for instance, a rectangle in which two of the sides are twice as long as the other two. What I call *general* concepts are concepts that are not maximally specific, for instance, the concept *rectangular*. The fact that a concept, such as *circular,* may be variously modified, such as *circular with a 1 m diameter* or *circular in France* does not mean that it is divisible into species, but I will not pause to try to define the difference between speciation and modification in this sense.

So suppose we take the high road and contend that the contents of our perceptions contain some general concepts. Then, in any given case, we can ask, which ones? Consider, for instance, your perception of a brown, wooden, Windsor armchair. What is the conceptual content of your perception? Here are some candidates: *That’s a chair. That’s a Windsor chair. That’s a wooden armchair. That’s a wooden piece of furniture*. These contents contain various, different, general concepts, at various criss-crossing levels of generality. Personally, I do not see how I could decide between them, not even if the perception were mine. Or consider your perception of a blue box with six rectangular sides. What is the conceptual content of your perception? Here are some candidates: *That is box-shaped. That is six-sided. That has six sides that meet at 90° angles. That is box-shaped and twice as long as it is wide.* Again, I do not see for myself how I could select among such candidates. So the high road poses a choice that I cannot make. The conclusion I draw is that it was a mistake to assume that the conceptual contents of perception include some general concepts.

When I pose this challenge and ask people to tell me specifically what the general concepts in the conceptual content of their perceptions might be, the answer I sometimes hear is that I am asking too much, because the conceptual content of perception is not something we need to be aware of, or conscious of, in such a way that we can tell, just like that, what the conceptual content of our perception is. My answer to this is threefold. First, the contention that the conceptual content of perception is unconscious should be especially challenging for anyone who connects, as many do (e.g. Pryor 2000; Glüer 2009), the contention that perceptions have conceptual content to the contention that perceptions justify beliefs. They would have to maintain that the justification that perceptions provide is one that a person cannot express in words or even reflect on. It would have to be a kind of “secret” justification. Second, the contention that the conceptual content of perception is unconscious will also be hard to bear for those who base their claim that perceptions have conceptual content on the contention that we sometimes *report* the content of our perceptions when we say things like, “That looks like a blue cube” (McDowell 1996, p. 165; Glüer 2009). If we can report the conceptual contents of our perceptions in this way, then the conceptual content is not *always* unconscious.^3^

Third, if perceptions really do have general concepts in their contents, then it should be possible to describe some experiments that would enable us to identify the conceptual content of a given perception. If we are supposed to choose between hypotheses, then we will need to be able to distinguish plausible hypotheses from implausible ones. So my challenge can be repeated at this level: Why is it any more plausible that the conceptual content of your perception is, say, *That’s a chair* than it is that it’s, say, *that’s a wooden piece of furniture*? Anyway, what kind of experiments would help here? They will not be experiments showing that the differences between objects falling under the same concept are perceived as *the same*. If the conceptual content of your perception is *that’s a chair*, then you will still be able to differentiate between a Windsor chair and a Hepplewhite chair. We might conceivably discover that we are perceptually more sensitive to certain differences than to others, but that will not show that the objects whose differences we are less sensitive to are classified in perception as belonging to the same kind. I cannot prove that no experiments could identify the conceptual content of a perception, but I am not aware of any serious attempt to describe experiments that could do it, and I cannot imagine any such experiments myself, and for these reasons I hold that skepticism is warranted.^4^

Another answer to my challenge that I sometimes hear is that I am falsely assuming that our language contains words that are appropriate to express the contents of perception. The reason why it is not very easy to pick out the right level of generality for the general concepts contained in our perceptions, it might be said, is that ordinary English lacks a suitable vocabulary. If this is not another statement of the objection I addressed in the previous two paragraphs, then I think it may be answered as follows: If your language lacks the requisite vocabulary, then you should be able to invent some vocabulary and teach it to me so that I can then understand what you say when you express the conceptual content of your belief. Even if I promise to wait and listen carefully, I doubt that you will be able to do that.

If the problem on the high road is that nothing distinguishes a given level of conceptualization as the level of the conceptual content of perception, then, it might be said, we can choose a level of conceptualization that is distinguished from all others by being maximally specific. This puts us on the low road. Our perceptions, it may be said, contain only maximally specific concepts, such as concepts of specific shapes of facing surfaces or concepts of specific shades of color. The problem with this road is that it is just not true. If I am looking at a rectangular blue slab, one might say that the specific shape that I see is the flat rectangular shape of the surface and a couple of the sides. But if we cut that slab in half and move the top half forward and shave a bit off the sides, then from a certain angle my visual experience of the two pieces might be indistinguishable from my visual experience of the undivided slab. I know of no reason to say that my experience represents the one facing surface rather than the other.

In answer to this challenge, someone might reply that there is in fact a reason to take the conceptual content to represent the solid slab rather than the divided slab, namely, that *normally* such a perception would be caused by a solid slab not a divided slab. It is very unlikely that the pair of slabs would be viewed in just such a way that they could not be distinguished from a solid slab. So if we happen to perceive two slabs as one, the mistake can be excused. But this answer takes us off the low road. The shape that is represented is supposed to be maximally specific. But there is literally an infinite variety of specific shapes and configurations that would cause a given perception. So the specific shape and configuration represented, supposing this is one, would be just one of an infinite number of possibilities. So its probability would be exactly zero. If the shape and configuration in question, the one that would normally cause such a perception, has a nonzero probability, then it must constitute a range and not be maximally specific.

It is the same with respect to color. I am assuming that if perceptions contain color concepts, then they are concepts of the real colors of things. As far as I know, all parties to the debate over perceptual representation assume that the properties that perceptions (typically) represent are properties that external objects really have. They are not properties of the perceptual or sensory experience itself. So on the low road the claim will be that a visual perception makes a claim to the effect that an object has a maximally specific objective color. But this is just not true. If you play around with a graphics program on your computer, you can create two brownish patches of color, one against a red background and one against a blue background, that appear to be exactly the same shade of brown, but which turn out to be very different colors when you take away the colored backgrounds (the one against the blue background being much greener than the one against the red background). Since the colors of the brown patches do not change when we place them against a uniform background, and they are clearly different colors when placed against a uniform background, they are different colors as well when placed against differently colored backgrounds, despite their looking the same. So when you are looking at the brown patches against the red and blue backgrounds, then since they look alike, although they are really different, your experience represents at most one of those patches as being the specific color it is. But since they look just alike, and the only difference in their presentation is the color of the background, there is no reason to say that the shade your perception represents is the one rather than the other.

Nor is it plausible that in this case neither color is correctly represented, although in other cases maximally specific colors are correctly represented. We would say that only if we assumed that the background color misleads us. But there is no reason to pick out one background color (e.g., some shade of white) and say that only against that background do we perceive colors as they really are. So if we want to say in the case described that neither perception correctly represents the color of the surface perceived, then we should deny that perceptions ever correctly represent colors at all. Since there is no adequate account of what specific color we are representing on the assumption that our perception represents a completely specific shade, we should conclude that in fact we do not represent any maximally specific shade. It is just not true that the concepts that make up the conceptual content of our perceptions include maximally specific color concepts.

As a final jab against the idea that perceptions have conceptual contents, I think it is fair to add that its status in the philosophical literature is that of a prejudice. The only widely recognized alternative is Peacocke’s idea that perceptions have *scenario content*, where a scenario content is roughly a *way of filling space* (Peacocke 1992). (Peacocke thinks that perceptions have conceptual content in addition.) But even that does not get as far as being a real alternative, because Peacocke says nothing at all about how a given perception in the head is associated with a particular scenario content. One finds very few other attempts to develop alternative conceptions of perceptual representation (exceptions would be Camp 2007, Rescorla 2009, and my own work, Gauker 2011, 2012). The theory that perceptual representations have conceptual content has not been compared to competitors and shown to be superior. For that reason, if for no other, there ought to be some receptivity to alternative accounts of perceptual representation.

## The Similarity Space Theory of Perceptual Representation

Now I will sketch a different hypothesis concerning perceptual representation. There are a lot of good questions that one could ask about this, but I will not try to answer all of them here, since my purpose in this paper is just to explain what one can say about the phenomena of seeing-as from this perspective. Actually, I think there are two fundamentally different aspects of perceptual representation, and only the second will play a role in my account of seeing-as. Except where noted, I will confine my attention to visual perception.

The first aspect of perceptual representation is the representation of spatial configuration. In visual perception we parse a scene into objects and surfaces. This involves distinguishing between individual wholes, representing the configuration of their parts, representing the orientation and shapes of surfaces and the orientation of major axes, and representing their spatial relations to one another. Pioneering studies of this kind of visual parse are Palmer (1978), Marr (1982) and Biedermann (1987) and basic research in this area is ongoing (e.g. Kubilius et al. 2014). The second aspect of perceptual representation will be a representation of similarity relations by means of a perceptual similarity space.

The representation of spatial configuration need not be thought of as conceptual. Rather, we can define the representation relation by means of a mapping of relations between representations and relations between things represented and a mapping between elements of the representation and elements of the scene represented. So we will have a relation, call it Π, such that for each relation *R* in a set of relations that hold between elements of the representation, Π(*R*) is a relation that holds between elements of the scene (for example, the relation of *being-to-the-left-of*). And we will have a function *h* such that, for each element *e* of the representation, *h*(*e*) is an element of the scene (such as a particular edge). If the mind literally contained maps, then Π could be a projection according to scale, but of course the mind does not literally contain maps, and so it cannot be that. We cannot demand that *h* be a homomorphism (with respect to Π), because we have to allow that spatial configuration can be misrepresented. But we can suppose that *h* is approximately a homomorphism, and we can suppose that Π and *h* can be identified teleologically, in terms of the role they play in promoting the success of the species that these functions pertain to in promoting the reproductive fitness of that species.

The other aspect of perceptual representation, which will be my focus here, can be modeled as a *perceptual similarity space*. My assumption is that perceptions can be modeled as *marks* in a perceptual similarity space. To explain what I mean, I need to start with the concept of an *objective quality space*. Objective quality space is a hyperspace with many, many dimensions that measure the location of an object along a variety of physical dimensions, namely, those that we are perceptually sensitive to. Distance in this similarity space (measured using the Pythagorean theorem) is inversely proportional to (perceptible) similarity. The dimensions of objective quality space will include not just the obvious ones, such as color (or its several dimensions), shape (its many dimensions), size, etc., but many others that less readily come to mind, such as jerkiness of motion. (The motion of a squirrel is jerkier than the motion of a cat.) There might be, for a given particular triangle, a dimension that measures degree of congruence to this particular triangle (and that dimension might be part of a subspace of dimensions that measure shape). Of course, many objects consist of a certain configuration of parts (e.g., a dining room furniture set, the parts of an animal). As I said, we will need a separate account of the perceptual representation of the configurations of parts. But given that, we can suppose that spatial arrangements can be arrayed along the dimensions of a objective quality space in ways that represent the similarities and differences between the configurations. Things composed of comparable parts can be more or less similar to one another with respect to the arrangement of those parts.

**Fig. 1:**
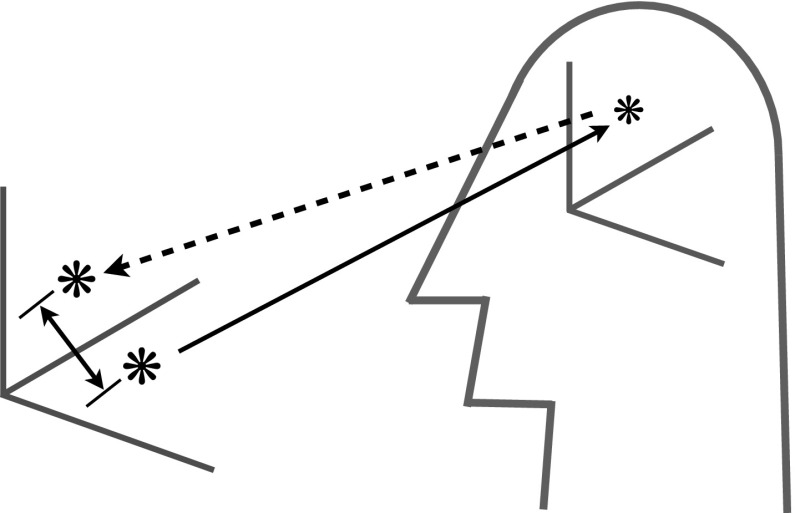
An objective quality space, a perceptual similarity space, a mark in perceptual similarity space that records the location of an object in objective quality space, and the point in objective quality space that the mark in perceptual similarity space corresponds to

Perceptual representations can be modeled by means of a perceptual similarity space, which is a hyperspace consisting of dimensions that correspond to the dimensions of objective quality space. (See Fig. 1.) An individual perception, in this model, can be conceived of as a *mark* in a perceptual similarity space. Exposure to an object causes a mark and the mark is a measure of the location of the object along each of a number of dimensions of objective quality space. However, we can distinguish between the location that an object is actually at in objective quality space and a point in objective quality space that the mark, caused by exposure to the object, *corresponds* to. The perception — the mark — will be *accurate* only to the extent that the corresponding point in objective quality space is near to the location where the object that is the cause of the mark actually is.

The next question would be: What is this correspondence relation? Roughly, the answer is: The point in objective quality space that corresponds to a mark in perceptual similarity space is *the* point in objective quality space at which the cause of the mark would have had to be located if the mark in perceptual similarity space had been recorded in the way that is biologically normal for the biological species to which the perceiver belongs. A further elaboration of this answer would take the form of an account of the biologically normal way of recording marks in perceptual similarity space, but I will not elaborate further here. (For elaboration, see Gauker 2011 or Gauker 2012.)

Perceptions, modeled as marks in a perceptual similarity space, are definitely not conceptual representations. Someone might think otherwise if he or she held that concepts themselves can be modeled as regions of a perceptual similarity space.^5^ In that case one might think that a mark in perceptual similarity space is a conceptualization of the object of perception as an *F* if the mark falls in the region that is the concept *F*. But it is an error to identify concepts with regions of perceptual similarity space. There are several good reasons to deny that concepts are regions of perceptual similarity space. Here is one: Suppose that some region of conceptual similarity space is my concept *bird*. Now imagine I encounter for the first time a certain sort of animal that I don’t recognize as a bird (an ostrich or a penguin perhaps). So my perception of it falls outside the region that is my concept *bird*. Then I learn that that animal is a bird (perhaps because someone tells me so, shows me the wings, etc.). In that case, the region that is my concept *bird* has to expand to include the new perception of a bird. Since my concept *bird* is the region and the region has expanded, my concept *bird* itself changed. But on the contrary, when I learned that the new thing was a bird, what I learned is that the very same concept, viz., my concept *bird*, which I formerly did not apply to that thing, applies to that thing. So the concept was never the region in the first place. (For elaboration and further objections, see Gauker 2007.)

It would also be a mistake to think that a perceptual representation, considered as a mark in perceptual similarity space, is a conceptualization on the grounds that measuring a thing’s location on any dimension of objective variability is already a conceptualization of it. When a thermometer measures the temperature of a room or of a human body, the reading on that thermometer is not already the subsumption of a particular under a concept. First of all, it does not refer to any particular enduring object, be it a room or a human body, and, second, it does not predicate of any object a general kind to which the object belongs. It does not do that even if it is hooked up to a machine, which, on the basis of the measurement, produces some genuine categorization. Similarly, a mark in perceptual similarity space, as I have described it here, is not in itself the subsumption of anything under a concept. Of course, the mind may use it to produce a genuine conceptualization of the object perceived.

In section 3, in questioning the assumption that perceptual representations have conceptual content, I remarked that it was not clear how one might go about identifying experimentally the content of a given perception. A similar question can be asked about the representation of perceptual representations in perceptual similarity space, namely, how could one identify the dimensions of perceptual similarity space? This is certainly not supposed to be open to introspection. However, one could operationalize in various ways judgments of comparative similarity. One operationalization would just consist in showing people pictures and then asking them questions of the form, “Is *x* more like *y* than like *z*?” Another operationalization would utilize priming effects. Does seeing *x* better prepare a participant to find *y* or better prepare the participant to find *z*? On the basis of these operationalizations, one could then hope to construct a list of three-place similarity “judgments” to the effect that *x is more like y than like z*. (I put “judgments” in quotation marks to indicate that these are not supposed to be conceptual thoughts.) Next, one could perform a multi-dimensional scaling to define a hyperspace in which *x* is closer to *y* than to *z* if and only if the judgment that *x is more like y than like z* is on the list. The last step would be to determine what the various dimensions of this space measure.

There is no guarantee that the method will work. It could turn out that the various operationalizations do not yield consistent results. Or it could turn out that every time we add a new object to the sample and generate the relative similarity “judgments”, the multi-dimensional scaling yields a new result (for example, requires an additional dimension). So my alternative conception of perceptual representation is nothing more than a hypothesis, which might turn out to be false, which is as it should be.

## Seeing-as

In terms of the model of perceptions as marks in perceptual similarity space I can now describe three different phenomena that could be described as nonconceptual forms of seeing-as.

### Ambiguity Resolution

Ambiguity resolution includes seeing Wittgenstein’s duck-rabbit as a duck or as a rabbit. It includes as well the perceptual choices that other such ambiguous figures, such as the Necker cube, present us with. It includes also seeing animals in clouds. It does not, however, include what is better described as “filling in”, such as seeing triangles in the Kanizsa triangles. That is probably better treated as a function of the perceptual representation of configuration. (Other terms might be preferred over “ambiguous” to describe these sorts of ambiguous figures, but this one is well-established in the literature.)

My hypothesis regarding the duck-rabbit drawing is that, precisely because it is so schematic, we have a degree of choice as to where we place a mark representing it in perceptual similarity space. We can place it closer to the other marks representing ducks, in which case we see it as a duck, or we can place it closer to the other marks representing rabbits, in which case we see it as a rabbit. The claim is not that placing it closer to the other marks representing ducks *causes* us to see it as a duck; rather, seeing it as a duck *is* nothing other than placing it closer to the marks representing ducks.

My claim is that we can exercise this choice without employing the concepts *duck* or *rabbit* at all. We have to have some perceptual representations of ducks and some of rabbits, but we do not need to conceive of the things so represented as ducks or as rabbits.^6^ Of course, if we have the concept *duck*, then having placed our perception of the duck-rabbit closer to the perceptions of ducks, we can say, and can think, *that’s a duck!* Moreover, the location of marks in perceptual similarity space may play a role in determining how concepts are applied.^7^ But that additional subsumption under a concept is a step beyond seeing-as as such.

So the claim is that perceptual ambiguity resolution is not an application of concepts. Against this claim, someone might object that the similarity comparisons that I am invoking require the application of concepts. At this point I am taking for granted, for the reasons given in section 3, that perceptual representations do not themselves contain concepts. So placing a mark in perceptual similarity space is not in itself an act of subsuming something under a concept. Still, it might be said, in choosing to place the mark representing a schematic drawing closer to the X’s rather than to the Y’s, one must inevitably exercise the concept *X* or the concept *Y*. The objection could be either that without the concepts *X* and *Y* one could not exercise a choice, or that the very act of measuring the distance between the mark representing the drawing and the marks representing the X’s and between the mark representing the drawing and the marks representing the Y’s necessitates an exercise of the concepts *X* and *Y*. In either case, I would like to try to persuade the reader that that is not so, by asking the reader to engage in a little experiment.

**Fig. 2:**
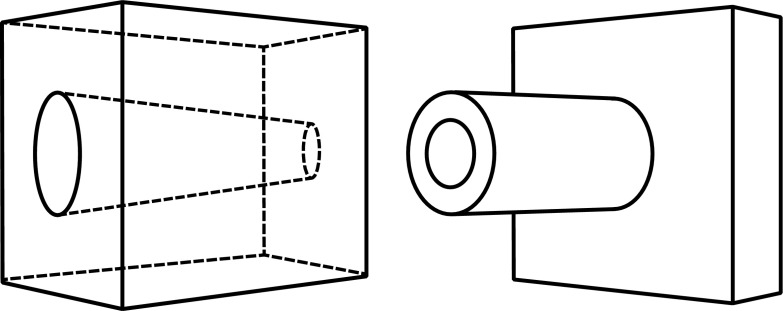
Two objects

Please examine the diagrams in Fig. 2. The figure on the left represents a box with a hole through it, the hole wider at one end than at the other. The figure on the right represents an object consisting of a tube coming out of a rectangular base. I think it is fair to assume that the reader does not subsume these objects under any generic concepts comparable to *duck* or *rabbit*. Not only does one’s perceptual representation not subsume them under any generic concept, but one does not immediately have any generic concept in mind at all. In particular, the reader does not have concepts of the maximally specific shapes of the objects depicted — because the drawings do not determine that much. Of course, the reader can *describe* these objects as I just did, for example, the one on the right as *an object consisting of a tube coming out of a rectangular base*. But, as we will see, that description does not provide a concept without which one could not represent the similarities between this object and others.

**Fig. 3:**
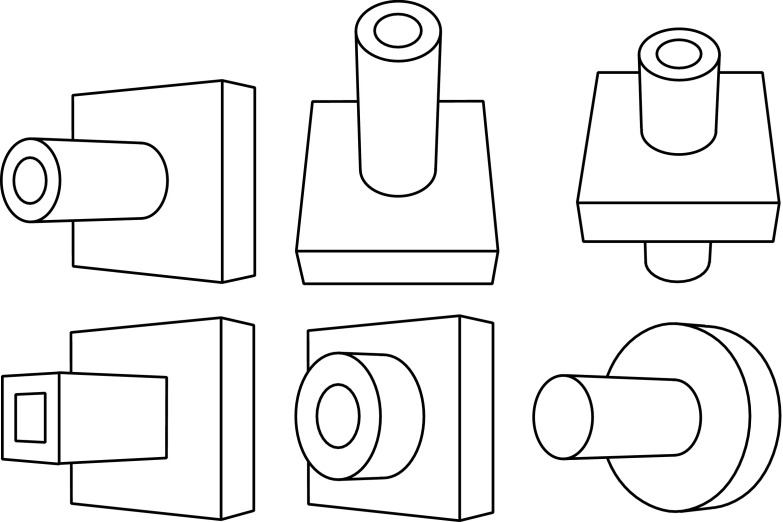
The original object is in the upper left

If the reader thinks that he or she has a generic concept of the kind of thing the object is, then I would challenge him or her to say which of the objects in Fig. 3 also fall under that genus. (Does it include the case in which the tube extends out of the back of the base? Does it include the case of the rectangular tube? Does it include the case of the squat tube?) My assumption is that, while the reader may undertake to “construct” a generic concept to which some of things and not others belong, by deciding which to include, it is not the case that the reader, upon observing the figure on the right in Fig. 2, already had a generic concept that, for each of these other figures, either did or did not cover it. That assumption goes as well for the description “tube coming out of a rectangular base”. If one wishes to refer to the object on the right-hand side of Fig. 2 and distinguish it from the object on the left, then one can come up with such a description, and that description does draw a distinction between some of the objects depicted in Fig. 3. But it was not the case that already in perceiving and representing the object on the right one had in mind such a description. Rather, the description is constructed with a view to distinguishing the object on the right from the object on the left for purposes of communication.

Notwithstanding the fact that the reader does not, as I will assume, have generic concepts of the kinds of things the objects in Fig. 2 are, I can still produce an ambiguous figure that the reader can *see as* either one of them. See Fig. 6 at the end of this article. Please look at that now and then come back. (I have placed Fig. 6 at the end of the article in the hope that the reader would not see it until directed to it.) So the drawing in Fig. 6 can be *seen as* either the box with a hole through it or as the tube sticking out of the base, despite the fact that the reader lacks generic concepts of the kinds of things that those objects belong to. The conclusion I draw is that the kind of seeing-as involved in the case of the duck-rabbit does not utilize concepts of genera.

### Heightened Discrimination

Another kind of seeing-as that might be explicated in terms of perceptual similarity spaces is what I call heightened discrimination. This is the effect on perception that comes with expertise. A person who does not know what a pine tree is and then learns to discriminate pine trees from deciduous trees might be thought to come to perceive pine trees differently than before.^8^ Likewise, the expertise that consists in being able to sort tree leaves into various kinds might influence the character of one’s perceptions of tree leaves. Or for a non-visual example, the ability to distinguish the sounds of an oboe, a bassoon and a clarinet might influence the character of one’s perception of the sound of an orchestra.

**Fig. 4:**
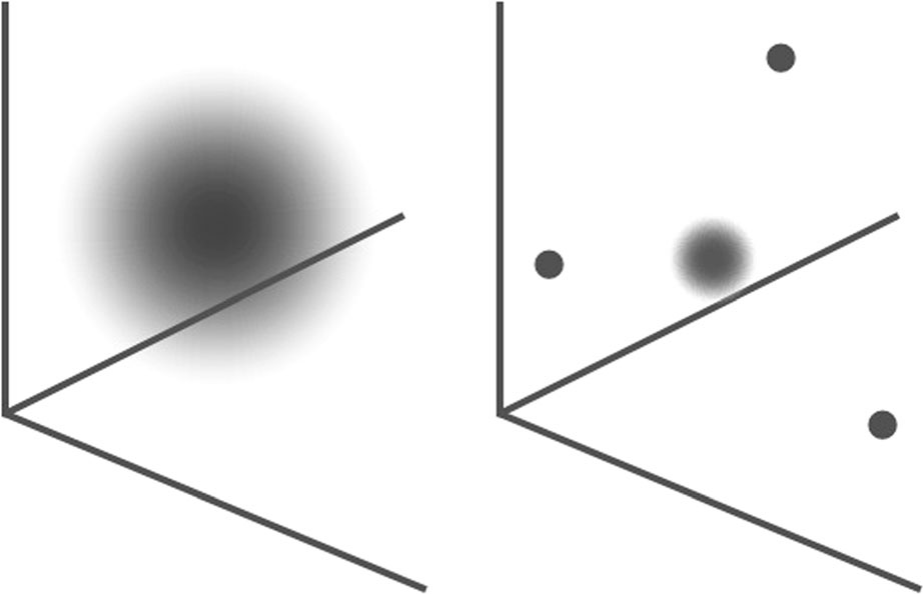
A fuzzily bounded region versus a more determinate mark

Here is one way of thinking about what happens to perception through the acquisition of expert powers of discrimination. I said that a perception can be modeled as a mark in a perceptual similarity space. A mark is an occupied *point* in perceptual similarity space. But actually perception might often be something more like a fuzzily bounded region in perceptual similarity space. We may think of this region as an imprecise measurement of the qualities of the object of perception along the various dimensions. (See Fig. 4.) However, we may suppose that once the mind has laid down a number of marks in a region of perceptual similarity space, the mind can produce more precise measurements of the location of objects in that region of perceptual similarity space. The already recorded marks may serve as landmarks in perceptual similarity space that allow measurement to proceed by comparisons to the locations of these other marks.

On this account, what happens when, through a long study of tree leaves, one acquires heightened powers to discriminate between tree leaves is that many different tree leaves are recorded in perceptual similarity space. Each different shade of green, each different variety of shape, each different kind of edge (smooth, lobed, serrated, etc.) and so on, serves to calibrate the scales for the dimensions that measure it. Thus, when a new tree leaf comes along, its location on each these dimensions can be more precisely pinpointed. The experience of taking in at a glance the tree leaf in all of its particulars and sharply distinguishing it from many others can be modeled as this capacity to pinpoint the location of the leaf in perceptual similarity space.

Again, I am taking for granted at this point that perceptions do not have conceptual contents (section 3). But as a way of checking this earlier conclusion, I want to point out the acquisition of expertise in perception does not lend itself to characterization in terms of concepts. That is because in many cases we can acquire such heightened powers of discrimination within a field of exemplars that exhibits a continuity of cases and in which it would be very hard to put meaningful labels on the different kinds. For example, I suppose that a student of modern painting could learn to reliably distinguish between paintings by Jackson Pollock and Jackson Pollock forgeries (of which there are many). But if one looks at a lot of Pollock paintings and Pollock forgeries (there are many photos of them on the internet), there does not seem to be any feature or disjunction of features that one can readily put into words that define the distinction. That is at least an initial reason to doubt that it is possession of a particular concept that enables the ability to discriminate.

One might contest this ground for doubt by claiming that in learning to distinguish between Pollocks and forgeries one acquires the concept *Jacksonpollockish* and this concept comes to inform one’s perceptions. But if one said this, one would be merely putting a label to an ability that one has no particular reason to describe as concept application. There was an evolution in Pollock’s work, from the early landscapes to his famous drip paintings, but even within the drip paintings, which is the kind most frequently forged, one acquires a sense of a range of possibilities within the genus that the forgeries lie outside of. One’s discriminatory ability might be grounded in a collection of paradigms, or one might form an impression of some central tendencies. The result might be described as a sense for the *Jacksonpollockish*, but nothing is added to the account by supposing that Jacksonpollockishness constitutes a kind representable in a concept.

### Dimension Addition

Perhaps there is a third kind of nonconceptual seeing-as, although I think ordinary experience does not reveal this as plainly as it reveals the first two kinds. Sometimes a particular object becomes very familiar to us. It might be the family dog. We come to recognize the dog in its many postures and moods. Or the object might be a favorite hand tool, such as a Swiss army knife with many blades and tools built into it. Or it might be a favorite sculpture by Jacques Lipschitz, a hard-to-grasp arrangement of shapes and surfaces. When this happens, we may be disposed to compare other objects we encounter to this familiar one. The familiar object might even become the origin, or zero, in a new dimension in perceptual similarity space. This dimension might be the product of collapsing several different dimensions into one: A dimension representing similarity to Fido sitting, a dimension representing similarity to Fido running, and so on, might be combined to produce a single dimension representing overall similarity to Fido.

When a new dimension is added to perceptual similarity space in this way, the relative similarity relations between objects is changed as well. In the old perceptual similarity space, it might be that three objects, *a*, *b* and *c*, are all equally distant from one another, forming the vertices of an equilateral triangle. But if a new dimension is added, and *a* and *b* have the same value on this new dimension, but *c* has a different value, then *a* and *b* may qualify as overall more like one another than either is to *c*.

**Fig. 5:**
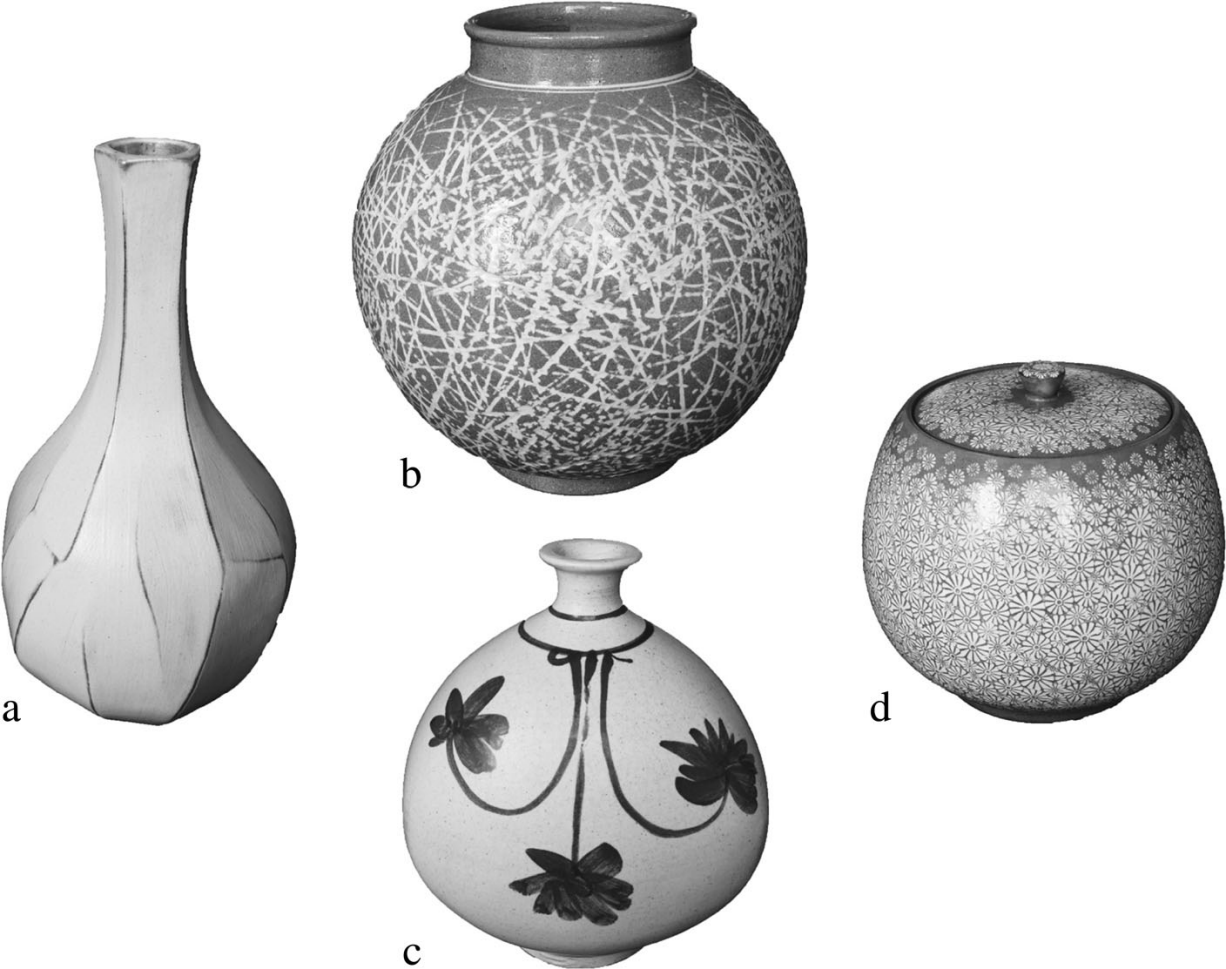
Four examples of Korean buncheong pottery.(**a**) by Yoo Byoung-Ho, (**b**) by Woochool, (**c**) by Lee Jong-Whan and (**d**) by Yu Yong-Cheol

For example, consider the four examples of Korean buncheong pottery in Fig. 5. If we just consider the three on the right (b, c and d), then we might think that they are all quite different from one another and that no two are more like one another than the third. But if the one on the left (a) is a familiar, cherished possession that forms the origin on a dimension of perceptual similarity space, then the two in the center (b and c) might appear to be more like one another than either is to the one on the right. That might be because the one on the right has a lid and none of the others do, or because the two in the center have in common with the one on the left a roughness in execution and an irregularity in pattern that is lacking in the one on the right.

**Fig. 6:**
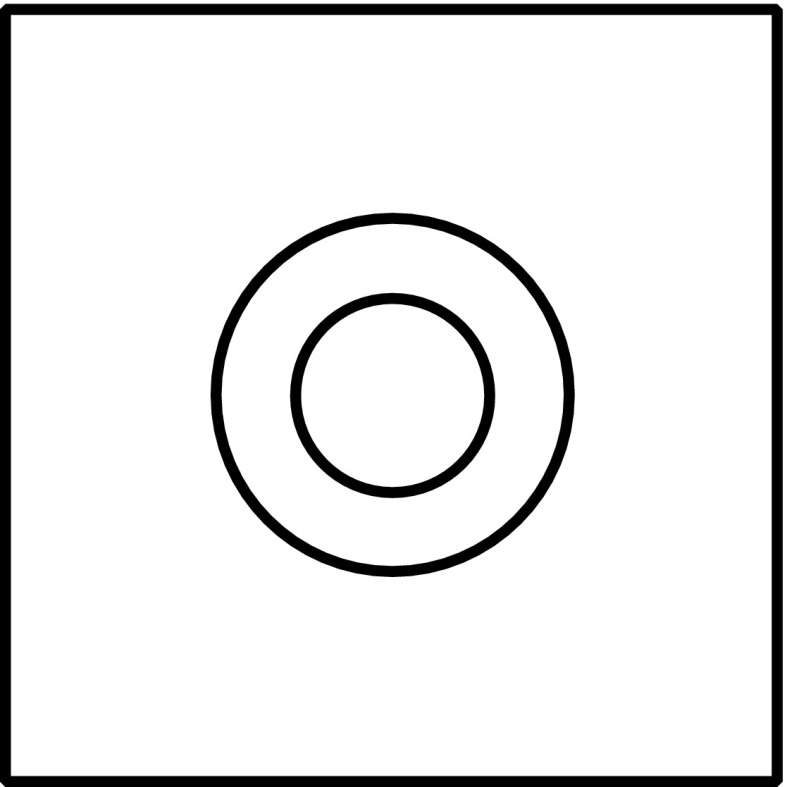
An ambiguous figure. (Compare Fig. 2)

In short, if it is true that particular, familiar objects can introduce new dimensions into perceptual similarity space, so that the relative positions of our perceptions in perceptual similarity space can shift, then in this way too experience can alter the character of our perceptual experience.

## Seeing-as, All the Time

The phenomenon of seeing-as has been appealed to by those who want to claim that all visual perception involves the subsumption of objects under general concepts. The idea is that the classic cases of seeing-as, such as the duck-rabbit, in one way or another make the conceptual content of our perceptions obvious, either by demonstrating the possibility of a substitution of the determining concept or by demonstrating the possibility of a refinement in perception through the acquisition of refined concepts. The conclusion to be drawn is that all visual perceptual representations have conceptual content; but the cases of so-called seeing-as announce the presence of that conceptual content very loudly.

Do my accounts of nonconceptual seeing-as likewise demonstrate that every act of visual perception is a case of seeing-as? On my account, the several forms of seeing-as are disparate; so they do not show that there is any one thing that happens in every act of visual perception. Visual perception on my account is not exclusively but always includes the placing of a mark in perceptual similarity space representing the location of an object, or arrangement of objects, in objective quality space. What I have called the phenomena of seeing-as are all different ways in which experience somehow affects the location of a mark in perceptual similarity space. In this light perhaps we should say that an infant’s first visual perceptions are not cases of seeing-as; it has to make do with the innate structures of perceptual similarity space alone. But certainly by the time a normal human reaches several years of age, all visual perceptions representing particular objects and scenarios are shaped in this way by past experience. So we should say, yes, all of the visual perceptions of a normal adult human being are cases of seeing-as.

## Footnotes

1 I will assume that readers are familiar with the duck-rabbit drawing from Wittgenstein’s *Philosophical Investigations* (1953). Reproductions can readily be found on the internet.

2 Elements of this conception of communication, and the accompanying conception of concepts, variously formulated, can be found in authors as diverse as Gottlob Frege (1892/1994), Jerry Fodor (1975), and David Lewis (1975) and Donald Davidson (1990). For a detailed critical exposition of this conception, see Gauker (2003).

3 Elsewhere I have challenged both the contention that such reports report the contents of perception and the contention that perceptions justify beliefs (Gauker 2017). However, these arguments do not drive the conceptual content of perception underground into the unconscious.

4 I have encountered the contention (but not in print) that the experimental demonstration of the categorial perception of phonemes (Liberman et al. 1957) and colors (Uchikawa and Shinoda 1996) proves that perception contains conceptual content. What is shown in these experiments is that discriminability and detectability is not evenly distributed over the physical variables. These experiments do not demonstrate that perceptions are representations that refer to a particular object and subsume that object under some general category and that can then serve as premises for inferences to other conceptual representations, such as judgments. Attempts to extend this experimental paradigm beyond shallow sensory qualities (e.g., Beale and Keil 1995; Todd et al. 2014) likewise do not show that perceptions themselves are conceptual representations, but the correct interpretation of these results is beyond the scope of this paper. Bermúdez 2003 proposes a strategy for identifying the conceptual contents of the thoughts of non-language-using creatures. It is questionable whether this method succeeds in pinning down a level of conceptualization, but in any case Bermudez provides no reason to think that the concepts he would attribute are specifically contents of perceptions.

5 Here I will pass over an issue that would arise for anyone who wanted to say that marks in perceptual similarity space are conceptual representations, namely, whether we want to say that the concept is itself a region of similarity space or want to say that the concept is the region of objective quality space to which the region of perceptual similarity space corresponds. The thesis that the concept is itself a region of perceptual similarity space does not sit happily either with the conception of concepts as elements of the content or with the conception of concepts as elements of the content-bearing representation.

6 Brewer (2007, 2011, p. 123) similarly proposes to explain the phenomenon of seeing the duck-rabbit as a duck as a matter of perceiving similarities between the duck-rabbit drawing and previously perceived ducks without presupposing that the perceiver needs to employ the general concept *duck* in order to do this. However, Brewer’s aim in his account is to avoid positing perceptual representations; that is no part of my aim. Another author who has argued that seeing-as does not require concepts is Orlandi (2011). However, her own positive account, according to which it is crucial that observers attend to different parts of the figure when seeing it in different ways, seems to be contradicted by empirical evidence (Toppino 2003).

7 For further discussion of how the location of marks in perceptual similarity space might guide conceptualization, see Gauker 2011.

8 This is an example that Susanna Siegel (2006) has used to try to argue that perceptions have conceptual content — or as she puts it, that kind properties are represented in perception.
